# Two cases of gallbladder torsion presenting with right lower quadrant pain: Clinical and imaging characteristics

**DOI:** 10.1097/MD.0000000000045535

**Published:** 2025-10-24

**Authors:** Xue-Wu Liu, Jun-Qiang Liang, Yuan-Yuan Wu, Nan Lei

**Affiliations:** a Department of Radiology, The People’s Hospital of Lezhi, Lezhi, China.

**Keywords:** acute abdomen, case report, gallbladder torsion

## Abstract

**Rationale:**

Gallbladder torsion (GT) is a rare cause of acute abdominal pain. Due to its rarity and nonspecific clinical presentation, GT is frequently misdiagnosed preoperatively. We report 2 cases of GT in patients initially suspected of acute appendicitis, both presenting with right lower abdominal pain. We conducted a retrospective analysis of the clinical and radiological findings in these 2 patients to enhance our understanding of and management for this condition.

**Patient concerns:**

Case 1 was a thin 80-year-old woman who was admitted to the hospital with migration of pain to the right lower quadrant, persisting for 3 days. Case 2 was a thin 84-year-old woman who was admitted to the hospital with right-sided abdominal pain that had persisted for over 20 hours.

**Diagnoses:**

Emergency abdominal ultrasound examination revealed a low-lying gallbladder in case 2 and an enlarged gallbladder with a small amount of perigallbladder fluid collection in both patients. Non-contrast computed tomography revealed an enlarged gallbladder with disrupted gallbladder mucosa in both cases. In addition, the first case exhibited a whirlpool structure in the cystic duct region, alongside a pedicled hyperdense structure located between the neck of the gallbladder and the liver bed. In the second case, a strip-like structure was observed within the cystic duct area. Postoperative pathology confirmed acute gangrenous cholecystitis in both patients’ gallbladders and acute simple appendicitis in their appendices.

**Interventions:**

Both patients underwent laparoscopic cholecystectomy.

**Outcomes:**

Both patients recovered well and were discharged within 1 week.

**Lessons:**

GT presenting with appendicitis-like symptoms is relatively uncommon. For thin elderly female patients presenting with signs related to appendicitis, the possibility of GT should be considered. Non-contrast computed tomography is valuable for diagnosing GT.

## 1. Introduction

Gallbladder Torsion (GT) is a rare condition characterized by the rotation of the gallbladder along its mesenteric axis, leading to compromised blood supply. The first documented case was reported by Wendel in 1898.^[1]^ To date, approximately 600 cases of GT have been recorded in the global literature.^[2]^ The preoperative diagnosis rate for GT remains notably low, with only 17% being reported in existing studies.^[3]^ Clinical manifestations are nonspecific and may include abdominal pain, nausea, vomiting, and symptoms mimicking acute cholecystitis.^[4]^ In this report, 2 cases of elderly female patients were diagnosed with GT via surgical confirmation at our institution. Both patients presented with right lower quadrant abdominal pain; 1 patient also experienced migration of pain to the right lower quadrant, mimicking the symptoms of acute appendicitis, a presentation that is rarely documented in previous case reports. We conducted a retrospective analysis of the clinical and imaging findings associated with these 2 cases to enhance understanding of this condition within clinical practice.

## 2. Case presentation

Case 1: An 80-year-old thin woman (BMI 15.2) was admitted to the hospital with a migration of pain to the right lower quadrant for 3 days. The patient denied chills, fever, nausea, vomiting, abdominal distension, diarrhea, or other associated symptoms. Upon physical examination, the clinician observed significant tenderness, rebound tenderness, and muscle rigidity localized to McBurney point in the right lower quadrant of the abdomen. Furthermore, Murphy sign was negative during the physical examination. At admission, laboratory results indicated that a white blood cell count of 14.17 × 10^9^/L (reference range: 3.5–9.5 × 10^9^/L) and the C-reactive protein level of 71.04 mg/L (reference range: 0–8 mg/L); both values were markedly elevated compared to normal ranges, suggesting an ongoing inflammatory response. Notably, liver function tests revealed no abnormal findings.

**Figure 1.**
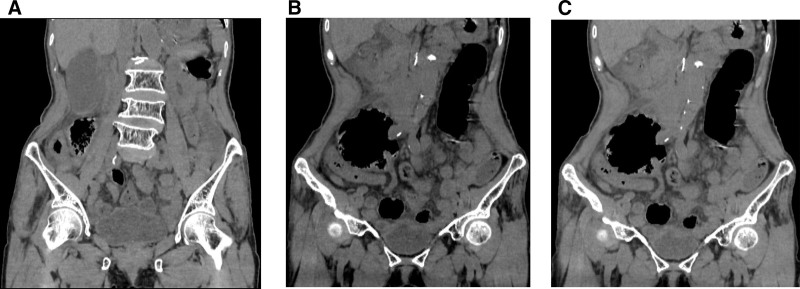
Case 1. (A) The gallbladder is enlarged, with a thickened and edematous gallbladder wall, and a linear slightly denser shadow along the inner wall; part of this shadow seems to float in the gallbladder cavity (the blue arrow). (B) “Hyperdense whirl sign.” A whirlpool structure was observed in both the neck of the gallbladder and its duct area (the red arrow), alongside a pedicled hyperdense structure located between the neck of the gallbladder and the liver bed (the yellow arrow). (C) The appendix in the right lower abdomen demonstrates mild thickening measuring 8 mm, accompanied by surrounding inflammatory exudation (the green arrow).

The clinician suspected acute appendicitis; however, differential diagnoses such as right adnexal torsion and right ureteral calculi could not be excluded. The patient subsequently underwent an emergency abdominal ultrasound (US), which revealed an enlarged gallbladder with no intraluminal calculi identified and a small pericholecystic fluid. This was followed by a non-contrast abdominal computed tomography (CT) scan, which revealed an enlarged gallbladder with thickened and edematous walls, as well as a linear slightly hyperdense shadow along the inner wall of the gallbladder; portions of this shadow appeared to be floating within the gallbladder lumen. No intraluminal calculi were identified, and increased density was observed in the pericholecystic fat stranding (Fig. 1A). Additionally, a whirlpool structure was observed in both the neck of the gallbladder and its duct area, alongside a pedicled hyperdense structure located between the neck of the gallbladder and the liver bed (Fig. 1B). Mild enlargement of the patient’s right lower abdominal appendix was noted, accompanied by minimal surrounding inflammatory exudation (Fig. 1C).

The patient subsequently underwent laparoscopic cholecystectomy. During surgery, a small volume of pale bloody inflammatory effusion was found in the abdominal cavity alongside an enlarged gallbladder exhibiting purplish-black gangrene and a complete 180° clockwise torsion of the cystic duct. No stones were detected within the gallbladder. During the surgical procedure, the attending surgeon identified congestion and edema of the appendix. Given the presence of secondary inflammatory changes, the decision was made to perform an appendectomy in addition to the primary procedure.

Postoperative pathology confirmed that acute gangrenous cholecystitis had developed in conjunction with acute simple appendicitis affecting both organs respectively. The patient recovered well and was discharged from the hospital 4 days later.

Case 2: An 84-year-old woman was admitted to the hospital due to the sudden onset of continuous but tolerable pain in the right lower abdomen for 20 hours. The patient was thin, but the BMI could not be accurately measured due to the inability to stand. The patient denied chills, fever, nausea, vomiting, abdominal distension, diarrhea, or other associated symptoms. Upon physical examination, the clinician observed mild abdominal muscle guarding and localized tenderness in the right lower quadrant, accompanied by mild rebound tenderness. No palpable enlargement of the gallbladder was noted in the right upper quadrant. Additionally, persistent yet tolerable pain was elicited upon palpation of the area below the right costal margin, consistent with a weakly positive Murphy sign. At admission, laboratory results indicated a white blood cell count of 12.46 × 10^9^/L (reference range: 3.5–9.5 × 10^9^/L) and the C-reactive protein levels of 48.06 mg/L (reference range: 0–8 mg/L); both values were markedly elevated compared to normal ranges, suggesting an ongoing inflammatory response. Notably, liver function tests revealed no abnormal findings.

**Figure 2.**
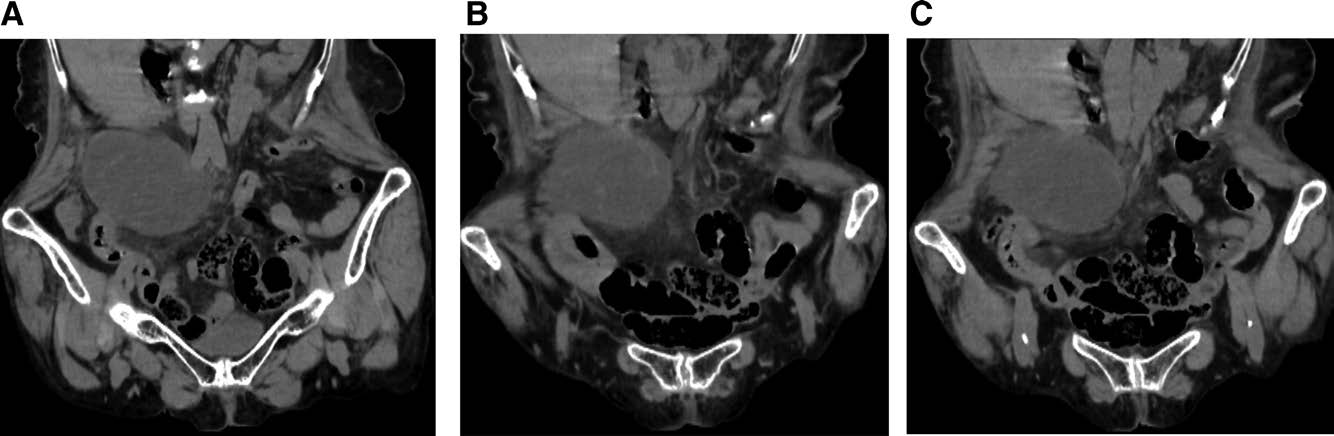
Case 2. (A) The gallbladder was not fixed to the liver bed, with an increased volume and thickened, edematous wall. A linear slightly denser shadow along the inner wall; part of this shadow seems to float in the gallbladder cavity (the blue arrow). (B) A strip-like structure with slightly increased density was observed in the region of the cystic duct (the red arrow), which may indicate torsion of the duct. The white arrow points to the common hepatic duct. (C) The appendix in the right lower abdomen demonstrates mild dilation measuring 9 mm, accompanied by surrounding inflammatory exudation (the green arrow).

The clinician suspected acute appendicitis; however, differential diagnoses such as cholecystitis and right ureteral calculi could not be excluded. The patient subsequently underwent an emergency abdominal US, which revealed a low-lying and enlarged gallbladder with no intraluminal calculi identified, and a small pericholecystic fluid. This was followed by a non-contrast abdominal CT scan, which revealed that the gallbladder was not fixed to the liver bed, with an increased volume and thickened, edematous wall. A slightly hyperdense shadow was observed on the inner wall of the gallbladder, with some areas appearing to float within it. No intraluminal calculi were identified, and increased density was observed in the pericholecystic fat stranding (Fig. 2A). A strip-like structure with slightly increased density was observed in the region of the cystic duct (Fig. 2B). Additionally, mild dilatation of the right lower abdominal appendix was noted along with surrounding inflammatory exudation (Fig. 2C).

The patient subsequently underwent laparoscopic cholecystectomy. During surgery, a small volume of dark brown inflammatory effusion was found in the abdominal cavity alongside an enlarged gallbladder exhibiting purplish-black gangrene and a complete 360° clockwise torsion of the cystic duct. No stones were detected within the gallbladder. During the surgical procedure, the attending surgeon identified congestion and edema of the appendix. Given the presence of secondary inflammatory changes, the decision was made to perform an appendectomy in addition to the primary procedure.

Postoperative pathology confirmed that acute gangrenous cholecystitis had developed in conjunction with acute simple appendicitis affecting both organs, respectively. The patient recovered well and was discharged from the hospital 1 week later. The timeline of the clinical course for the 2 cases is shown in Table 1. This study adheres to CARE guidelines for reporting.^[5]^

**Table 1:** Timeline of clinical course for 2 cases.

	Timeline	Medical event	Results/notes
Case 1	3 d preadmission	Symptom onset	Migration of pain to the right lower quadrant, persisted for 3 days
	Admission day	Physical examination	Tenderness at McBurney point
		Laboratory tests	White blood cell: 14.17 × 10^9^/L, C-reactive protein: 71.04 mg/L
		Abdominal ultrasound	Enlarged gallbladder, pericholecystic fluid
		Non-contrast computed tomography	Enlarged gallbladder, a whirlpool structure, “hyperdense whirl sign,” and enlarged appendix
		Laparoscopic cholecystectomy and appendectomy	180° clockwise cystic duct torsion, gangrenous gallbladder, and inflamed appendix
		Postoperative pathology	Acute gangrenous cholecystitis and acute simple appendicitis
	4 d after admission	Discharge	Recovered well
Case 2	20 h preadmission	Symptom onset	Continuous pain in the right lower abdomen, persisted for 20 h
	Admission day	Physical examination	Tenderness at McBurney point and a weakly positive Murphy sign
		Laboratory tests	White blood cell: 12.46 × 10^9^/L, C-reactive protein: 48.06 mg/L
		Abdominal ultrasound	Low-lying and enlarged gallbladder, pericholecystic fluid
		Non-contrast computed tomography	Displaced gallbladder, a strip-like structure, and enlarged appendix
		Laparoscopic cholecystectomy and appendectomy	360° clockwise cystic duct torsion, gangrenous gallbladder, and inflamed appendix
		Postoperative pathology	Acute gangrenous cholecystitis and acute simple appendicitis
	1 wk after admission	Discharge	Recovered well

## 3. Discussion

GT is a rare cause of acute abdomen that can occur at any age, although it is most commonly observed in female patients aged between 60 and 80 years.^[6]^ The etiology remains unclear. Aging and reduced body weight may lead to loss of adipose tissue supporting the gallbladder, decreased mesenteric elasticity, and visceral organ displacement. These factors, combined with spinal deformities causing organ drooping, are potential predisposing factors.^[7]^

The symptoms and signs associated with GT are nonspecific; clinical manifestations often mimic those of acute cholecystitis. During physical examination, clinicians may note a positive Murphy sign along with a palpable mass in the right upper quadrant. However, when the gallbladder is displaced from its normal anatomical bed or when inflammation around the gallbladder extends to other regions within the abdominal cavity, clinical presentations may vary somewhat. For instance, our first patient exhibited initial symptoms akin to those of acute appendicitis—specifically metastatic pain localized in the right lower quadrant—which aligns with previously reported symptoms associated with GT.^[8]^ In contrast, our second patient experienced pain primarily in her right lower quadrant due to her gallbladder being positioned outside its typical location and closer to this area. Furthermore, laboratory tests for GT tend to be nonspecific. Only approximately half of affected patients demonstrate elevated inflammatory markers such as white blood cells and C-reactive protein; notably, liver function tests are typically normal among most patients.^[3]^ In our study cohort, both reported patients presented with elevated white blood cell counts and C-reactive protein levels while maintaining normal liver function parameters.

The clinical features and laboratory tests of GT are not specific, making the preoperative diagnosis heavily reliant on imaging examinations. Abdominal radiography is ineffective for diagnosing GT. Abdominal US can identify gallbladder distension, fluid accumulation around the gallbladder, and detachment of the gallbladder from its bed. Furthermore, it has been reported that abdominal US may detect lengthening and/or torsion of the gallbladder pedicle, which appears as a conical structure^[9]^; however, no subsequent reports confirm whether this sign is beneficial in diagnosing GT. Contrast-enhanced CT and magnetic resonance imaging (MRI) better demonstrate twisted pedicle.^[10–12]^ Although the literature indicates that the number of patients with GT undergoing MRI before surgery is limited, the diagnostic accuracy of MRI can reach up to 60%.^[3]^ Magnetic resonance cholangiopancreatography is undoubtedly considered the most effective radiological examination for diagnosing GT before surgical intervention.

However, in emergency settings, abdominal US and non-contrast CT are frequently utilized as the preferred imaging modalities. In the case we reported, the patient underwent abdominal US and non-contrast CT. Although a preoperative diagnosis of GT was not confirmed, certain imaging features provided critical insights for its diagnosis.

Abdominal US revealed that case 2 exhibited a low position of the gallbladder. Additionally, both patients demonstrated an enlarged gallbladder volume along with a small amount of fluid surrounding the gallbladder, indicating inflammatory changes within this organ. In Figure 1B, the non-contrast CT revealed a whirlpool structure at the neck of the gallbladder and in the cystic duct area, suggesting torsion of the cystic duct. In Figure 2A, the CT revealed a “floating gallbladder” sign. This finding suggests that the gallbladder had been displaced from its anatomical bed as a result of mesenteric membrane torsion. Additionally, in Figure 2B, a strip-like structure with slightly increased density was observed in the cystic duct region, which indicates torsion of the duct. The 2 patients exhibited distinct imaging manifestations of cystic duct torsion. The observed variations in imaging manifestations may be attributed to differences in the degree of cystic duct torsion or to anatomical variability of the cystic duct. Furthermore, in Figures 1A and 2A, both patients exhibited thickening of the gallbladder wall accompanied by fragmentation and discontinuity of mucosal layers—findings suggestive of ischemia and gangrene within the gallbladder tissue. Notably, it has been observed that non-contrast CT scans in patients with GT can reveal a central high-density core in the pedicle with a twisted appearance that can be seen between the gallbladder neck and liver bed, which the researchers have termed the “hyperdense whirl sign.”^[13]^ This sign can also be observed in our Figure 1B image, which might become a characteristic imaging manifestation. The investigators propose that the detection of such high-density cores on non-contrast CT may often indicate thrombus formation within mesenteric structures associated with the gallbladder—a hypothesis yet to be definitively validated at this time.

## 4. Limitations

By conducting a retrospective analysis of the non-contrast CT of 2 patients with GT, we identified several characteristic manifestations. Nevertheless, given the small sample size, our conclusion might be limited. We aim to provide diagnostic clues for clinicians and radiologists. We anticipate that more case reports regarding GT will emerge in the future to validate these manifestations.

## 5. Conclusion

GT presenting with appendicitis-like symptoms is relatively uncommon. For thin elderly female patients presenting with signs related to appendicitis, the possibility of GT should be considered. Non-contrast CT is valuable for diagnosing GT. Enlargement or displacement of the gallbladder, fragmented or discontinuous mucosa, and torsion of the cystic duct are important signs suggesting GT. The “hyperdense whirl sign” has a specificity, but more cases are still needed for verification. Although GT is a rare clinical disease, increased awareness and accumulation of case data can improve preoperative diagnosis.

## Author contributions

**Conceptualization:** Xue-Wu Liu, Jun-Qiang Liang, Yuan-Yuan Wu, Nan Lei.

**Data curation:** Jun-Qiang Liang.

**Investigation:** Jun-Qiang Liang.

**Resources:** Xue-Wu Liu.

**Supervision:** Jun-Qiang Liang, Yuan-Yuan Wu, Nan Lei.

**Writing – original draft:** Xue-Wu Liu.

**Writing – review & editing:** Jun-Qiang Liang, Yuan-Yuan Wu, Nan Lei.
